# Association between fat-soluble vitamins and self-reported health status: a cross-sectional analysis of the MARK-AGE cohort

**DOI:** 10.1017/S0007114521004633

**Published:** 2022-08-14

**Authors:** Caroline Sarah Stokes, Daniela Weber, Stefan Wagenpfeil, Wolfgang Stuetz, María Moreno-Villanueva, Martijn E. T. Dollé, Eugène Jansen, Efstathios S. Gonos, Jürgen Bernhardt, Beatrix Grubeck-Loebenstein, Simone Fiegl, Ewa Sikora, Olivier Toussaint, Florence Debacq-Chainiaux, Miriam Capri, Antti Hervonen, P. Eline Slagboom, Nicolle Breusing, Jan Frank, Alexander Bürkle, Claudio Franceschi, Tilman Grune

**Affiliations:** 1 Department of Molecular Toxicology, German Institute of Human Nutrition, 14558 Potsdam-Rehbrücke, Germany; 2 Food and Health Research Group, Faculty of Life Sciences, Humboldt-Universität zu Berlin, 14195 Berlin, Germany; 3 NutriAct-Competence Cluster Nutrition Research Berlin-Potsdam, Nuthetal 14458, Germany; 4 Institute of Medical Biometry, Epidemiology and Medical Informatics, Saarland University, Homburg, Germany; 5 Department of Food Biofunctionality, Institute of Nutritional Sciences (140), University of Hohenheim, 70599 Stuttgart, Germany; 6 Molecular Toxicology Group, Department of Biology, University of Konstanz, 78457 Konstanz, Germany; 7 Human Performance Research Centre, Department of Sport Science, University of Konstanz, 78457 Konstanz, Germany; 8 Centre for Health Protection, National Institute for Public Health and the Environment, PO Box 1, 3720 BA Bilthoven, The Netherlands; 9 National Hellenic Research Foundation, Institute of Biology, Medicinal Chemistry and Biotechnology, Athens, Greece; 10 BioTeSys GmbH, Schelztorstr. 54-56, 73728 Esslingen, Germany; 11 Research Institute for Biomedical Aging Research, University of Innsbruck, Rennweg, 10, 6020 Innsbruck, Austria; 12 UMIT TIROL – Private University for Health Sciences, Medical Informatics and Technology, 6060 Hall in Tyrol, Austria; 13 Laboratory of the Molecular Bases of Ageing, Nencki Institute of Experimental Biology, Polish Academy of Sciences, 3 Pasteur street, 02-093 Warsaw, Poland; 14 URBC-NARILIS, University of Namur, Rue de Bruxelles, 61, Namur, Belgium; 15 Department of Experimental, Diagnostic and Specialty Medicine, Alma Mater Studiorum, University of Bologna, Bologna, Italy.; 16 Interdepartmental Center - Alma Mater Research Institute on Global Challenges and Climate Change, University of Bologna, Bologna, Italy; 17 Medical School, University of Tampere, 33014 Tampere, Finland; 18 Section of Molecular Epidemiology, Leiden University Medical Centre, Leiden, The Netherlands; 19 Department of Applied Nutritional Science/Dietetics, Institute of Nutritional Medicine, University of Hohenheim, Stuttgart 70599, Germany; 20 Department of Experimental Pathology, University of Bologna, Bologna, Italy; 21 German Center for Diabetes Research (DZD), 85764 München-Neuherberg, Germany; 22 German Center for Cardiovascular Research (DZHK), Partner Site Berlin, 13347 Berlin, Germany; 23 University of Potsdam, Institute of Nutritional Science, Nuthetal, Germany; 24 University of Vienna, Department of Physiological Chemistry, Faculty of Chemistry, 1090 Vienna, Austria

**Keywords:** *α*-tocopherol, Micronutrients, Plasma, Retinol, Vitamin D

## Abstract

Self-rated health (SRH) is associated with higher risk of death. Since low plasma levels of fat-soluble vitamins are related to mortality, we aimed to assess whether plasma concentrations of vitamins A, D and E were associated with SRH in the MARK-AGE study. We included 3158 participants (52 % female) aged between 35 and 75 years. Cross-sectional data were collected via questionnaires. An enzyme immunoassay quantified 25-hydroxyvitamin D and HPLC determined *α*-tocopherol and retinol plasma concentrations. The median 25-hydroxyvitamin D and retinol concentrations differed significantly (*P* < 0·001) between SRH categories and were lower in the combined fair/poor category *v*. the excellent, very good and good categories (25-hydroxvitamin D: 40·8 *v.* 51·9, 49·3, 46·7 nmol/l, respectively; retinol: 1·67 *v.* 1·75, 1·74, 1·70 µmol/l, respectively). Both vitamin D and retinol status were independently associated with fair/poor SRH in multiple regression analyses: adjusted OR (95 % CI) for the vitamin D insufficiency, deficiency and severe deficiency categories were 1·33 (1·06–1·68), 1·50 (1·17–1·93) and 1·83 (1·34–2·50), respectively; *P* = 0·015, *P* = 0·001 and *P* < 0·001, and for the second/third/fourth retinol quartiles: 1·44 (1·18–1·75), 1·57 (1·28–1·93) and 1·49 (1·20–1·84); all *P* < 0·001. No significant associations were reported for *α*-tocopherol quartiles. Lower vitamin A and D status emerged as independent markers for fair/poor SRH. Further insights into the long-term implications of these modifiable nutrients on health status are warranted.

Preserving optimal health and quality of life is becoming vital, particularly in light of the expected increase in global life expectancy, and hence the ageing population^(1,2)^. Accordingly, strategies to reduce the projected future burden on healthcare systems are urgently required^(2)^. Health status is influenced by a multitude of genetic, environmental and lifestyle factors, with nutrition playing a pivotal role^(3,4)^.

Fat-soluble micronutrient deficiencies have, for instance, been associated with various diseases such as cancer, diabetes and CVD^(5–10)^. The specific fat-soluble vitamins A, D and E have a wide array of functions, including important immunomodulatory and inflammatory, and antioxidant-related processes, and deficiencies in these vitamins can contribute to, amongst others, the weakening of the immune system^(5,8,11,12)^. Indeed, low micronutrient status, as measured in plasma, has not only been linked to specific diseases as mentioned above, but also to poorer general health status^(13–15)^. For instance, low plasma *α*-tocopherol concentrations (a marker for vitamin E status) in older adults have been associated with poor physical and mental health and with greater inflammation^(14)^. In addition, two longitudinal cohorts, the Chinese Longitudinal Healthy Longevity Survey (CLHLS) and US National Health and Nutrition Examination Surveys (NHANES) observed older adults to have lower serum 25-hydroxyvitamin D concentrations when their self-rated health (SRH) status was categorised as poor^(13)^.

Inadequate micronutrient status, can, to a certain extent, be attributed to the ageing process, given that age has been reported as an independent predictor and main contributor to fat-soluble micronutrient status^(16)^. Nutritional status, however, declines not only with increasing age but also in the setting of chronic health conditions or in those following poor diets, which are age-independent. Indeed, a recent analysis from the 2005–2016 NHANES including 26 282 adults^(17)^ illustrated widespread dietary deficiencies in fat-soluble micronutrients. The following proportions of the population were reported to be below the estimated average requirement for vitamins A, D and E: 45 %, 95 % and 84 %, respectively^(17)^. Depending on the local fortification strategies, vitamin D status will of course be strongly influenced by sun exposure^(18)^.

Lower nutrient status has been linked to a higher risk of death, as evidenced in a meta-analysis for vitamin D^(19)^ and in a longitudinal study with 15 years of follow-up, which associated both poor diets and poor SRH with increased mortality^(20)^. Of note, SRH has recently been reported to be a significant predictor of mortality in participants with no chronic conditions^(15)^. On account of the biological functions of the fat-soluble vitamins A, D and E, we hypothesised that lower plasma concentrations of these essential micronutrients are a prevalent risk factor for poorer health status, irrespective of age. Thus, the aim of this cross-sectional analysis was to assess for associations between plasma concentrations of fat-soluble vitamins A, D and E and self-reported health status in 3158 subjects aged between 35 and 75 years, participating in the European multi-country MARK-AGE study. A previous study using this cohort has demonstrated that self-reported health could act as a marker distinguishing people at risk of becoming frail, therefore associating self-reported health status with adverse health outcomes^(21)^.

## Participants and methods

### Particpants

Data were analysed from the cross-sectional MARK-AGE population study, which aimed to identify a set of biomarkers of ageing^(22–24)^. For this analysis, 3158 participants recruited from 8 European centres were included. These participants were divided into three main groups. The RASIG group consisted of age-stratified individuals from the general population. This group comprised 2310 women and men aged 35–75 years. This age range, together with the ability to provide written informed consent, represented the main criterion for inclusion.

Descendants from nonagenarians (people aged between 90 and 99 years) were recruited into the second study group. These 537 men and women were offspring of nonagenerian subjects who had been recruited in another study, the GEHA study (Genetics of Healthy Aging^(25)^). Thus, this second group was named GO (GEHA offspring). Thirdly, 311 spouses from the recruited GO participants formed the SGO group (spouses of GO), and these subjects were enrolled as a lifestyle control group. Recruitment of GO and SGO participants was carried out in Belgium, Finland, Greece, Italy, the Netherlands and Poland. The exclusion criteria for the MARK-AGE study have been described^(23)^ and included seropositivity for HIV or for the hepatitis B or C viral infections, current treatment for cancer or with glucocorticoids, < 50 % of lifetime spent in country of residence, and inability to provide informed consent.

### Ethics and study procedures

The Local Research Ethics Committees of the respective recruiting centres provided ethical approval for the MARK-AGE project, which conformed to the Declaration of Helsinki and which was registered retrospectively at the German Clinical Trials Register (DRKS00007713). All participants provided written consent prior to participation. The recruitment together with the data collection took place in the following centres: Tirol/Innsbruck (Austria), Namur (Belgium), Esslingen (Germany), Athens, and other nearby regions (Greece), Bologna (Italy), Warsaw (Poland), Tampere (Finland), and Leiden (the Netherlands). Between November 2008 and June 2012, trained nurses and physicians collected the participant data, for which the standard operating procedures have been published previously^(26)^; therefore, only the measurements that are relevant for the present analysis are described below.

### Health status

Health status was assessed using a standard self-report five-point scale for rating phsyical health, which included the following options for the question in general, would you say your health is excellent, very good, good, fair or poor. This scale is frequently used in self-perceived health statistics which asks participants to provide a subjective assessment of their general health^(27)^. Participants also answered questions about falls and hospitalisation in 12 months preceding participation, current medication intake and the presence of past and current medical problems at the time of the survey.

As per Reichmann *et al.* ^(28)^, a co-morbidity index was subsequently computed by counting the total number of self-reported current health conditions. The conditions included elevated blood pressure or cholesterol, heart-related conditions (e.g. angina and heart failure), autoimmune diseases, diabetes, thyroid disease such as hypothyroidism or hyperthyroidism, osteoporosis, arthritis, liver or kidney-related conditions, respiratory conditions, neurological conditions, memory-related or mental health conditions, vision or hearing impairments, chronic pain such as back or leg pain. The total number of co-morbidities were grouped into three levels: 0–1, 2–3 and 4+. For this variable, missing values were coded as if the condition was present (worst-case scenario) and included in the analyses.

### Sociodemographic and lifestyle data

Questionnaire-based data captured sociodemographic factors^(21–23)^ such as age, sex, marital status, family history, lifestyle behaviours such as nutritional intake, smoking habits and alcohol consumption, and eduction level. Education was documented based on a seven-point scale and divided into three groups: (1) don’t know, never attended school and elementary school unfinished; (2) elementary school finished, first or second stage secondary level, and third-level education; and (3) university education. BMI data were used that had been calculated from relevant anthropometry (height and weight), which also included waist cirumference. Data from blood pressure measurements were also included.

### Fat-soluble vitamins

Plasma measurements of three fat-soluble vitamins were chosen for inclusion: serum 25-hydroxyvitamin D, which is the accepted status marker for vitamin D. For vitamin A, retinol and for vitamin E, *α*-tocopherol were analysed, since they represent the greatest proprtion of these vitamins in blood. HPLC was used with UV and fluorescence detection to simultaneously measure tocopherols and retinol. For internal quality control, the inter-batch CV for *α*-tocopherol and retinol were 6·3 % and 3·7 %, respectively. The full details on sample preparation and chromatographic conditions have been published^(16)^. Plasma 25-hydroxyvitamin D was quantified using an enzyme immunoassay (OCTEIA, AC57F1, IDS, Boldon, UK)^(24)^. The inter-assay variation was 4·7 % as determined in five analysis cohorts with two or three quality control samples. The intra-assay variation was 1·7 % as determined in one assay with eight samples in the first row and eight samples in the last row of the microtiterplate.

### Study outcomes and statistics

The primary outcome was to assess for associations between fat-soluble vitamins (A, D and E) and SRH. The Kolmogorov–Smirnov test was used to ascertain the data distributions, and depending on the outcome, either mean ± standard deviation or median (with interquartile range) was used to report participant characteristics, results and to guide the appropriate statistical tests. Given the very small number of participants in the poor health status group (1·3 % of the cohort), this category was combined with the fair category – a procedure used by others^(15,29,30)^. One-way ANOVA or the Kruskal–Wallis test was used to assess differences between the four categories of health status and the continuous variables (age, BMI, waist cirumference, blood pressure and fat-soluble vitamin concentrations), followed by the *t* test or Mann–Whitney U test, respectively, with Bonferroni correction. The fat-soluble vitamins were subsequently grouped into quartiles and, in the case of vitamin D, also into accepted cut-offs determining vitamin D status, and were then compared, alongside other categorical variables (sex, BMI classification, smoking status, alcohol consumption status, falls, hospital stays over the past year, medications, co-morbidities and season) with the categories of health status using Pearson’s *χ* ^2^ test. As there is no universal consensus regarding accepted cut-offs for vitamin D status, the following were applied to this analysis based on guideline recommendations and widespread usage in research studies: serum 25-hydroxyvitamn D ≥ 75 nmol/l (optimal/normal), between 74 and 50 nmol/l (insufficiency), between 49 and 30 nmol/l (deficiency), and < 30 nmol/l (severe deficiency)^(31,32)^. Health status was compared between the three groups of participants included, however given the larger variation in age range for the RASIG group (35–75 years) compared with the GO and SGO groups (mainly 55–75 years), subgroup analyses stratified by age with participants ≥ 55 years only were carried out (depicted in the flow chart in the supplementary materials).

Univariate and multiple logistic regression analysis (with cumulative logit) was used to explore determinants of health status, with results presented as OR and corresponding 95 % CI. The univariate analysis included the following variables: the standard cut-offs for vitamin D, in addition to the quartiles for retinol and *α*-tocopherol and adjustments for the following possible confounders: age (categorised into four age groups: 35–44, 45–54, 55–64 and 65–75 years), sex, education (categorised into three groups: elementary school unfinished, school finished and higher education), marital status (four groups: married, divorced, widow and never married), BMI (as covariate), season of blood collection (categorised into the four seasons), co-morbidities (categorised into three groups based on number of co-morbidities: 0–1, 2–3 and 4+), study group (three categories: RASIG, GO and SGO), supplement intake (yes and no), hospital stays in preceding year (yes and no), smoking status (never, previous and current) and status of alcohol consumption (abstainer, consumer). The dependent variable for the ordinal logistic regression consisted of four categories: excellent, very good, good and fair/poor, as frequently used in other studies and national surveys^(29,30)^. Only the significant variables from the univariate analysis were entered into the model for multiple logistic regression. The same procedure (using the same independent variables) was carried out for univariate and multiple binary logistic regression. The dependent variable was dichotomised by grouping the fair/poor health status categories and compared with the excellent/very good/good categories, as has been recommended^(33)^ and used in comparable studies^(34,35)^. All statistical analyses were carried out with SPSS 25.0 (IBM). Statistical significance was determined with two-sided *P*-value ≤ 0·05. We followed the STROBE guidelines for cross-sectional studies for reporting the results. No participant was excluded based on missing data, given the very low proportion of missing values (indicated in the tables).

## Results

### Participant characteristics

In total, 3158 MARK-AGE participants (52 % women, median age 60 (IQR: 50–66) years) were included in this analysis. The characteristics of the participants stratified by category of health status are presented in Table 1. Participants with self-perceived health status rated as fair or poor were older than those rating health status as excellent or very good. They also had higher BMI and waist cirumference, with fewer participants having BMI in the normal range (< 25 kg/m^2^) and a larger proportion in the obese range (≥ 30 kg/m^2^). Moreover, the proportion of non-smokers in the fair/poor group was lower than the other health status categories, but percentage of alcohol abstainers was higher. Additionally, the rate of hospitalisation and falls over the past 12 months was higher, and percentage of participants with no co-morbidities or not taking any medication was lower. All the above differed significantly.

**Table 1.** Baseline participant characteristics stratified by self-rated health status (Number and percentages)§

	All 3158 (100)		Excellent 369 (11·4)		Very good 1112 (35·2)		Good 1292 (40·9)		Fair/poor 385 (12·2)		*P*
	*n*	%	*n*	%	*n*	%	*n*	%	*n*	%	
Sociodemographic data											
Sex, F/M	1654/1504	52·4/47·6	170/199	46·1/53·9	570/542	51·3·0/48·7	705/587	54·6/45·4	209/176	54·3/45·7	**0·023**
Age (years)											
Median	60		57		57		60		63		**< 0·001**
IQR	50–66		45–65^a^		47–65^a^		52–67^b^		57–68^c^		
Anthropometry											
BMI (kg/m^2^)											
Median	26		25		25		26		28		**< 0·001**
IQR	23–29		23–27^a^		23–28^b^		24–29^c^		25–31^d^		
< 25 kg/m^2^	1331	42·2	203	55·0	531	47·8	485	37·6	112	29·1	**< 0·001**
25–30 kg/m^2^	1238	39·2	142	38·5	423	38·0	533	41·3	140	36·4	
≥ 30 kg/m^2^	588	18·6	24	6·5	158	14·2	273	21·1	133	34·5	
Waist circumference (WC) (cm)											
Median	92		89		90		94		98		**< 0·001**
IQR	84–100		80–96^a^		83–98^b^		85–102^c^		87–105^d^		
Systolic blood pressure (mm Hg)											
Median	133		130		131		134		138		**< 0·001**
IQR	120–147		120–142^a^		120–145^a^		122–149^b^		125–151^b^		
Diastolic blood pressure (mm Hg)											
Median	80		80		80		80		80		**0·010**
IQR	74–88		72–85^a^		74–89^b^		74–89^a^		73–90^a^		
Lifestyle factors											
Non-smoker*	2626	83·2	319	86·4	964	86·7	1052	81·4	291	75·6	**< 0·001**
Alcohol abstainer	455	14·4	33	8·9	118	10·6	209	16·2	95	24·7	**< 0·001**
Factors related to health status											
Hospitalisation†	355	11·0	26	7·0	103	9·3	150	11·6	76	19·7	**< 0·001**
Falls‡	344	10·9	28	7·6	100	9·0	142	11·0	74	19·3	**< 0·001**
No co-morbid conditions	1014	32·1	210	56·9	442	39·7	327	25·3	35	9·1	**< 0·001**
No medications taken	1400	44·3	233	63·1	604	54·3	491	38·0	72	18·7	**< 0·001**
Season of blood collection											
Winter	687	21·8	89	24·1	257	23·1	289	22·4	52	13·5	**< 0·001**
Spring	664	21·0	65	17·6	208	18·7	285	22·1	106	27·5	
Summer	784	24·8	84	22·8	276	24·8	314	24·3	110	28·6	
Autumn	1023	32·4	131	35·5	371	33·4	404	31·3	117	30·4	
Plasma fat-soluble vitamins											
25-hydroxyvitamin D (nmol/l)											
Median	47·4		51·9		49·3		46·7		40·8		**< 0·001**
IQR	36·2–60·7		41·2–67·5^a^		37·7–62·6^b^		35·5–59·1^c^		31·5–52·4^d^		
*α*-Tocopherol (µmol/l)											
Median	28·2		28·2		28·4		28·3		27·3		0·344
IQR	23·9–33·4		23·8–33·5		23·9–33·2		24·1–33·6		23·2–32·6		
Retinol (µmol/l)											
Median	1·72		1·75		1·74		1·70		1·67		**< 0·001**
IQR	1·45–2·02		1·50–2·08^a^		1·50–2·02^a^		1·43–2·01^b^		1·36–1·99^b^		

Significant *P*-values are highlighted in bold. Significant results for Kruskal–Wallis pairwise comparisons indicated by a different superscript letter (i.e. sharing same superscript indicates no statistically significant difference).

* The ‘non-smoker’ category represents current non-smokers and includes those who were previous smokers.

† Hospitalisation was defined as being hospitalised (with an overnight stay) within the last 12 months.

‡ Falls was defined as one or more falls in the last 12 months.

§ The following variables contained missing values (ordered as presented in table): BMI (*n* 1), hospitalisation status (*n* 6), 25-hydroxyvitamin D (*n* 140) and retinol (*n* 136).

#### Fat-soluble vitamins

**Fig. 1.**
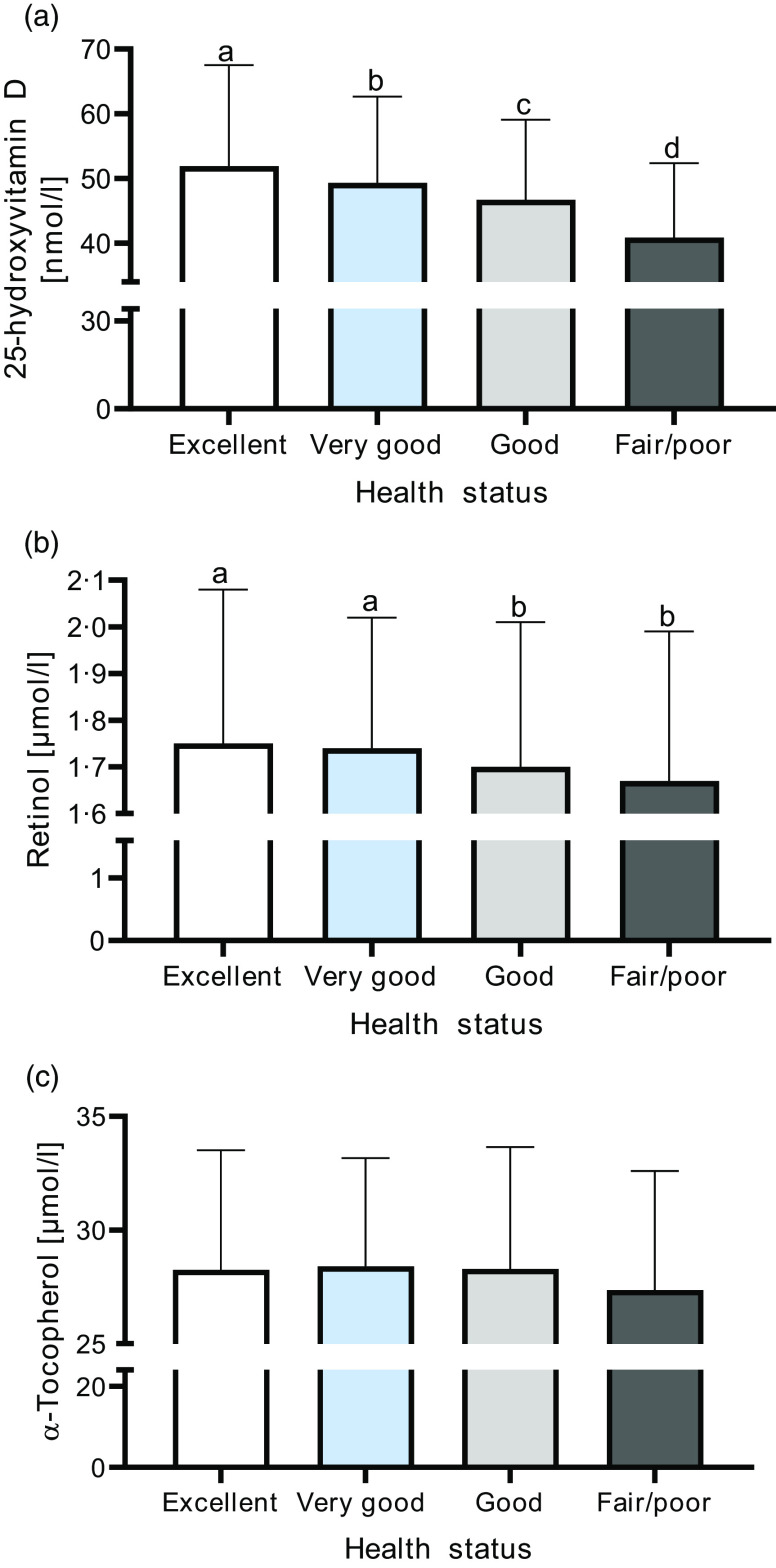
Median and interquartile range for plasma 25-hydroxyvitamn D (a), retinol (b) and *α*-tocopherol (c) based on self-reported health status. ^a,b,c,d^ Bars with different superscript letters indicate that their medians differ significantly from the others in that figure; conversely, bars sharing a common superscript letter illustrate that their medians are not significantly different from each other (*P* < 0·05), as analysed using Kruskal–Wallis with pairwise comparisons and Tukey’s *post hoc* test. Health status: excellent (*n* 369); very good (*n* 1112; good (*n* 1292); fair/poor (*n* 385).

**Fig. 2.**
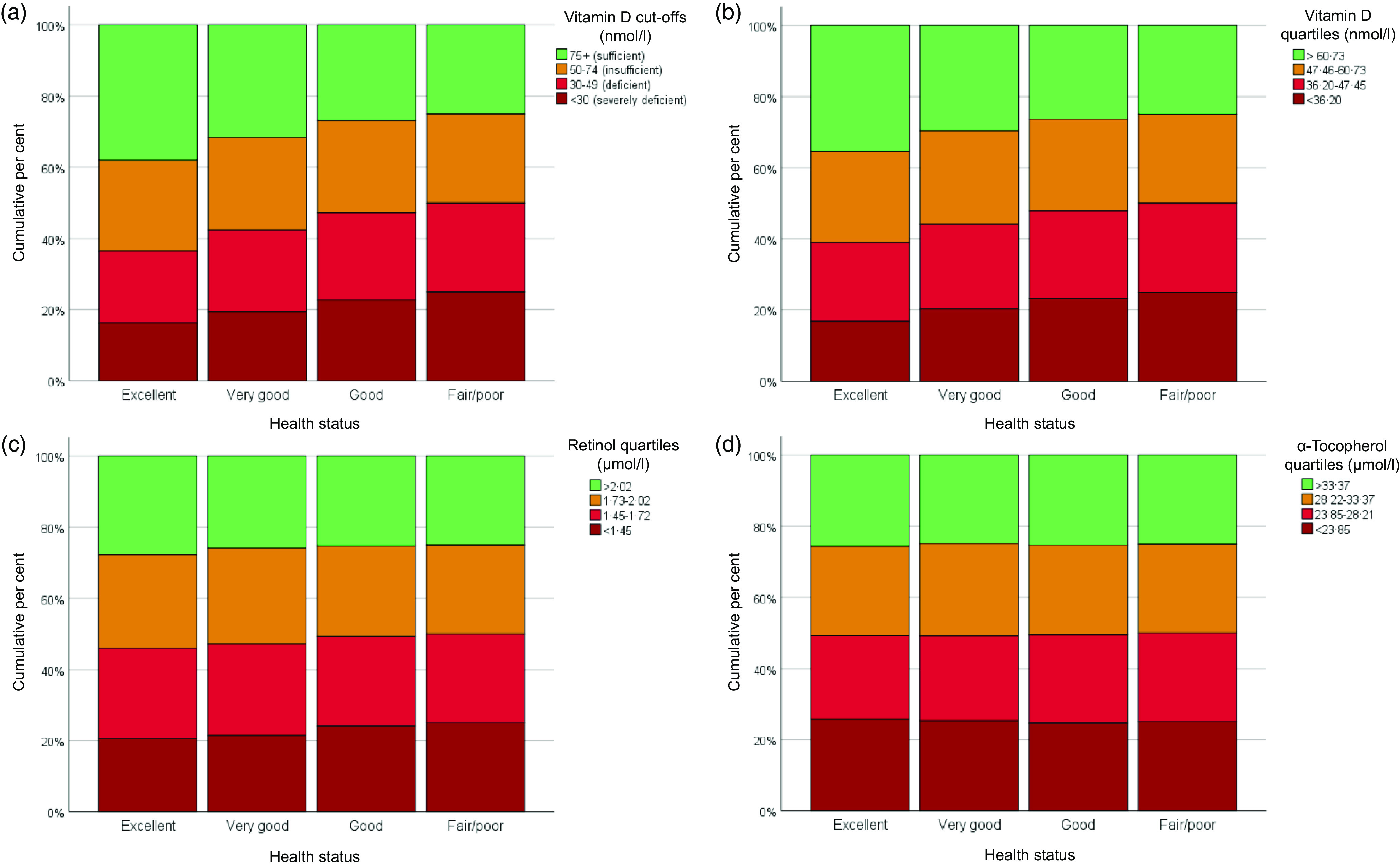
When comparing the proportion of participants in the different vitamin D groups with the categories of self-reported health status, a statistically significant association was demonstrated between categories for vitamin D status: *χ* ^2^(9) = 78·97, *P* < 0·001 (a) and for vitamin D quartiles: *χ* ^2^(9) = 84·49, *P* < 0·001 (b). Retinol quartiles also illustrated a significant association with category of health status: *χ* ^2^(9) = 23·36, *P* < 0·0005 (c) but *α*-tocopherol quartiles did not: *χ* ^2^(9) = 6·11, *P* = 0·729 (d).

Plasma 25-hydroxyvitamin D and retinol concentrations were significantly lower in participants that rated health status as fair/poor as compared with the excellent, very good and good ratings (vitamin D) and excellent and very good ratings (retinol; see Fig. 1(a) and (b)). No significant differences were observed for *α*-tocopherol (Fig. 1(c)). SRH was also compared with the fat-soluble micronutrients after grouping them into quartiles and in the case of vitamin D, into the accepted cut-offs representing vitamin D status: optimal/normal (25-hydroxyvitamin D ≥ 75 nmol/l), insufficient vitamin D status (between 74 and 50 nmol/l), deficient (between 49 and 30 nmol/l) and severely deficient (< 30 nmol/l)^(31,32)^. There was a statistically significant association between vitamin D cut-off and health status category (*χ* ^2^(9) = 78·97, *P* < 0·001); however, the association was small (Cramer’s V = 0·093). A similar association was reported for vitamin D quartiles (*χ* ^2^(9) = 84·49, *P* < 0·001; Cramer’s V = 0·097). Specifically, a higher proportion of participants with lower vitamin D status also had a lower rating for health status (Fig. 2(a) and (b)). As shown in Fig. 2(c) and (d), retinol quartiles were also significantly associated with category of health status (*χ* ^2^(9) = 23·36, *P* < 0·0005; Cramer’s V = 0·051), whereas *α*-tocopherol quartiles were not (*χ* ^2^(9) = 6·11, *P* = 0·729; Cramer’s V = 0·026). Specifically, a higher proportion of participants with lower retinol status also reported a more unfavourable health status.

The influence of season on vitamin D status was illustrated in this cohort, with a significant difference between four seasons, when comparing plasma 25-hydroxyvitamin D concentrations in the entire cohort using the Kruskal–Wallis test (Summer: 58 nmol/l, Autumn: 58 nmol/l, Winter: 44 nmol/l and Spring: 47 nmol/l, *P* < 0·001). Furthermore, summer and winter seasons were also compared (but not spring or autumn, as they do not represent extremes in or lack of sun exposure in all included countries) using the categories for vitamin D cut-offs. These *χ* ^2^ tests corroborated the aforementioned results for vitamin D. When analysing summer season separately, a significant difference in the proportion of participants in the different vitamin D categories was still observed for the four groups of health status (*P* = 0·002 and *P* < 0·001 for vitamin D status and quartiles, respectively). A similar result was also observed when comparing winter season (*P* = 0·002 and *P* < 0·001, respectively). Neither retinol nor *α*-tocopherol were significantly associated with SRH category based on winter and summer seasons (all *P* > 0·05).

#### Study groups

Self-reported health status was also evaluated based on the three groups of study participants: RASIG: *n* 2310 (73·1 %); GO: *n* 537 (17·1 %); and SGO: *n* 311 (9·8 %). Supplementary Table 1 summarises the number (and percentage) of participants in these groups based on the categories of SRH, which differed significantly (*χ* ^2^(4) = 41·49, *P* < 0·001; Cramer’s V = 0·081). The GO group had the highest percentage of participants rating health status as excellent (14·2 %), and the SGO had the highest percentage for self-reported fair/poor health status (14·8 %). Given, that the RASIG group included participants from the age of 35 years, a subgroup comparison between the three groups only including participants ≥ 55 years confirmed the above findings (*χ* ^2^(4) = 28·53, *P* < 0·001; Cramer’s V = 0·84),

#### Country of participation

Supplementary Table 2 summarises the proportion of participants in the four categories of SRH based on country. The highest percentage of participants reporting fair/poor health status was from Poland (26·8 % in all ages and 33·7 % in those ≥ 55 years), with the lowest reported from Austria with 1·5 % (2 % in ≥ 55 years). Health status category was significantly associated with country, with similar results obtained when stratifying the cohort according to age and including those 55 years and older (both *P* < 0·001).

### Vitamin D status associated with dichotomised self-reported health status

To ascertain the effects of fat-soluble vitamins on the likelihood that participants reported fair or poor health status, binary logistic regression was performed; hence, health status categories were dichotomised as follows (excellent, very good, good *v*. fair and poor). The univariate analysis assessed vitamin D status, retinol and *α*-tocopherol quartiles as well as the following confounders: sex, age group, education, marital status, BMI, smoking and alcohol consumption status, supplement use, number of current co-morbidities, number of medications, hospital visits during the preceding 12 months, season of blood sampling, country of residence and study group. Only the significant variables were included in the multiple binary regression analysis. Of these seventeen predictor variables tested in the univariate analysis, thirteen were statistically significant (as shown in online Supplementary Table 3) and included in multiple binary logistic regression: vitamin D status, retinol quartiles, age, BMI, education, marital status, smoking status, alcohol consumption status, number of current co-morbidities, number of medications, hospital visits during the preceding 12 months, season and country of residence. No multicollinearity was found among the independent variables.

The multiple logistic regression model was statistically significant (*χ* ^2^(34) = 639·78, *P* < 0·001). The model explained 36·9 % (Nagelkerke R2) of the variance in self-reported health status and correctly classified 89·6 % of cases. Three of the thirteen independent variables included were not significantly associated with self-perceived fair/poor health status: age, season and retinol quartiles (Table 2). Thus, from the three fat-soluble vitamins, only vitamin D status demonstrated a significant association with SRH, where for example, participants with a severe vitamin D deficiency (plasma 25-hydroxyvitamin D < 30 nmol/l) had 2·07 higher adjusted odds of reporting fair/poor health status than those with more favourable vitamin D status (*P* = 0·02).

**Table 2.** Logistic regression analysis for self-reported health status as the dependent variable (Odd ratio and 95 % confidence intervals)†

Independent variables	Binary regression		Ordinal regression		Independent variables	Binary regression		Ordinal regression	
	OR	95 % CI	OR	95 % CI		OR	95 % CI	OR	95 % CI
Sex (reference: female)			0·97	0·83, 1·13	Hospitalised past 12 months	1·72	1·21, 2·44**	1·35	1·08, 1·69**
Age group: 35–44 (years)	Reference		Reference		Season: Summer	Reference		Reference**	
45–54 (years)	1·28	0·69, 2·37	0·98	0·82, 1·17	Autumn	1·09	0·72, 1·63	1·22	0·98, 1·51
55–64 (years)	1·38	0·79, 2·48	0·92	0·74, 1·15	Winter	1·02	0·67, 1·57	1·37	1·12, 1·67**
65–75 (years)	1·37	0·75, 2·51	1·24	0·96, 1·61	Spring	1·38	0·91, 2·12	1·49	1·20, 1·84***
Education: Higher education	Reference**		Reference***		Vitamin D status (nmol/l): Optimal (≥ 75)	Reference***		Reference**	
Finished school	1·66	1·22, 2·27**	1·16	0·54, 2·52	Insufficiency (74–50)	0·83	0·46, 1·47	1·33	1·06, 1·68**
Elementary school unfinished	3·31	1·16, 9·47*	1·78	0·81, 3·88	Deficiency (49–30)	1·21	0·68, 2·13	1·50	1·17, 1·93**
Marital status: married	Reference		Reference**		Severe deficiency (< 30)	2·08	1·11, 3·90*	1·83	1·34, 2·50***
Divorced	1·03	0·70, 1·51	0·52	0·35, 0·76**	Retinol quartiles (µmol): First (> 2·02)	Reference		Reference***	
Widow	0·71	0·42, 1·19	0·75	0·54, 1·04	Second (2·02–1·73)	0·89	0·61, 1·31	1·44	1·18, 1·75***
Never	1·65(	1·04, 2·62*	0·72	0·50, 1·04	Third (1·72–1·45)	1·01	0·69, 1·47	1·57	1·28, 1·93***
BMI (kg/m^2^)	1·04	1·01, 1·07**	0·96	0·94, 0·97***	Fourth (< 1·45)	1·45	0·99, 2·12	1·49	1·20, 1·84***
Alcohol (reference: no)	0·63	0·45, 0·87**	0·75	0·61, 0·93**	Country: Germany	Reference***		Reference***	
Smoking status: Never	Reference**		Reference***		Austria	0·20	0·08, 0·54**	1·13	0·78, 1·63
Previous	1·12	0·82, 1·51	1·44	1·15, 1·78**	Belgium	0·93	0·49, 1·76	0·70	0·52, 0·94*
Current	1·88	1·32, 2·69**	1·71	1·39, 2·10***	Finland	2·73	1·43, 5·21**	1·11	0·78, 1·58
Medications: 0	Reference ***		Reference ***		Greece	2·18	1·16, 4·09*	0·28	0·20, 0·39***
1–2	1·28	0·88, 1·87	1·70	1·25, 2·30**	Italy	1·37	0·74, 2·54	2·07	1·50, 2·85***
3–4	2·64	1·74, 4·01***	3·16	2·37, 4·20***	The Netherlands	0·91	0·40, 2·10	2·70	1·90, 3·83***
5+	4·69	4·69, 7·37***	4·02	2·98, 5·42***	Poland	4·36	2·47, 7·69***	1·04	0·74, 1·45
Number of co-morbidities: 0–1	Reference ***		Reference***		Study group: RASIG	Reference		Reference*	
2–3	1·58	0·98, 2·54	2·86	2·37, 3·45***	GO	–		1·45	1·09, 1·92*
4+	5·86	3·66, 9·39***	5·05	4·05, 6·31***	SGO	–		1·35	1·01, 1·80*

Significant *P*-values:

* *P* = 0·05–0·01;

** *P* = 0·01–0·001;

*** *P* < 0·001.

† Ident variables included in multiple binary logistic regression analyses: vitamin D status (four categories), retinol quartiles (four categories), age group (four categories), BMI, education (three categories, marital status (four categories), smoking status (three categories), alcohol consumption status, number of current co-morbidities (three categories), number of medications (four categories), hospital visits during the preceding 12 months, season of blood sampling (four categories) and country of residence (eight categories). Independent variables included in multiple ordinal logistic regression analyses: vitamin D status (four categories), retinol quartiles (four categories), sex, age group (four categories), BMI, education (three categories, marital status (four categories), smoking status (three categories), alcohol consumption status, number of current co-morbidities (three categories), number of medications (four categories), hospital visits during preceding 12 months, season of blood sampling (four categories), country of residence (eight categories) and study group (three categories). The following variables contained missing values (ordered as presented in table): BMI (*n* 1), hospitalisation status (*n* 6), 25-hydroxyvitamin D (*n* 140), retinol (*n* 136).

### Association of both vitamin A and D with categories of self-reported health status

Ordinal logistic regression was performed to determine the effect of the three fat-soluble vitamins on the four groups of SRH (excellent, very good, good and fair/good): as with binary logistic regression, the following predictor variables were assessed in a univariate analysis: sex, age group, education, marital status, BMI, smoking and alcohol consumption status, supplement use, number of current co-morbidities, number of medications, hospital visits during the preceding 12 months, season of blood sampling, vitamin D status, quartiles for retinol and *α*-tocopherol, country of residence and study group. Only variables with significant associations were included in the multiple ordinal regression analysis. As with the binary logistic regression, no multicollinearity existed among the independent variables.

The results of the univariate analysis are reported in Supplementary Table 4 and of the multiple regression analysis in Table 2. Overall, fifteen of the seventeen predictor variables were included in the multiple regression analysis, apart from supplement use and *α*-tocopherol, because they did not show significant associations with SRH. Vitamin D was significantly associated with SRH. The adjusted OR of being in a lower category of health status (i.e. reporting fair/poor health) for those with insufficient vitamin D levels or with vitamin D deficiency *v*. optimal vitamin D status was 1·33 (95 % CI, 1·06, 1·68) and 1·50 (95 % CI 1·17, 1·93), respectively. Both these results were statistically significant (*P* = 0·01 and *P* = 0·001, respectively). Participants with severe vitamin D deficiency (< 30 nmol/l) displayed a significantly higher odds of reporting a lower category of health status (adjusted OR 1·83, 95 % CI, 1·34, 2·50, *P* < 0·001) than those with optimal plasma vitamin D concentrations.

Additionally, participants in all three quartiles for retinol showed significantly (all *P* < 0·001) higher odds of reporting a lower category of SRH as compared with those in the first quartile (unlike the binary regression results), specifically, adjusted OR 1·44, 95 % CI, 1·18, 1·75 (second quartile), adjusted OR 1·57, 95 % CI, 1·28, 1·93 (third quartile) and adjusted OR 1·49, 95 % CI 1·20, 1·84 (fourth quartile).

The results for other predictor variables (Table 2) influencing SRH demonstrated that the number of co-morbidities was associated with a higher odds of reporting lower health status, the odds of which increased with a higher level of co-morbidities when compared with none or having only one co-morbidity. As expected, medication intake (*v*. no medications) yielded a higher odds for reporting lower health status. Both season and country influenced the odds of reporting a lower health status, specifically spring and winter seasons and the Netherlands and Italy.

## Discussion

Poor diets have been associated with a number of non-communicable diseases and adverse health outcomes^(36)^, and both poor nutrition and poor SRH have been associated with an increased risk of death^(19,20)^. The data from the current analysis suggest that SRH is significantly associated with plasma vitamins A and D status. Specifically, both plasma retinol and 25-hydroxyvitamin D but not *α*-tocopherol were associated with SRH, confirming our hypothesis that low plasma concentrations of fat-soluble vitamins are risk factors for self-perceived poorer health status, irrespective of age.

Ordinal logistic regression analysis demonstrated a higher odds of reporting fair/poor health when plasma vitamin D concentrations were below clinically defined optimal concentrations, supporting previous findings^(37)^. Moreover, participants in the lowest category representing a severe vitamin D deficiency (< 30 nmol/l (< 12 ng/ml)) had the highest odds, with the OR (adjusted OR 1·83; 95 % CI 1·34, 2·50) being comparable with that of another cross-sectional study assessing vitamin D concentrations and SRH (OR 1·72; 95 % CI 1·16, 2·54)^(37)^. Binary logistic regression analysis corroborated the findings for the severe vitamin D deficiency category. In another study focussed on young/middle-aged healthy adult males, serum 25-hydroxyvitamin D was significantly associated with SRH in multiple regression analysis (OR 0·91; 95 % CI 0·85, 0·97; *P* = 0·004). Specifically, every 2·5 nmol/l (1 ng/ml) increase of serum 25-hydroxyvitamin D was associated with 9 % reduction in the odds of reporting fair or poor SRH^(38)^. Moreover, Rafiq *et al.* reported participants in the lowest category of serum 25-hydroxyvitamin D concentrations (< 25 nmol/l) to have a lower odds (OR 0·50, 95 % CI 0·33, 0·76) of scoring a higher SRH compared with participants with vitamin D concentrations between 25 and 50 nmol/l (OR 0·88, 95 % CI 0·67, 1·15), a finding that was reported independently of chronic conditions^(39)^. Additionally, a recent analysis on vitamin D and SRH which combined the current MARK-AGE cohort with the Health 2000 cohort reported the association between vitamin D status and SRH to exist regardless of presence or absence of diseases^(15)^. In another study, lower vitamin D concentrations (assessed as continuous variable) were mildly associated with lower SRH^(34)^. Of note, however, mean 25-hydroxyvitamin D concentrations were higher than the reported median concentrations in our cohort (59·5 *v.* 47·4 nmol/l). Similar associations were observed in two longitudinal cohorts, the CLHLS (China) and NHANES (USA) where older adults with SRH categorised as poor had lower plasma 25-hydroxyvitamin D concentrations^(13)^.

When compared with the highest (first) quartile for retinol, the three quartiles representing lower retinol concentrations displayed increased odds of fair/poor SRH. No significant associations were observed for retinol with binary logistic regression analysis. To the best of our knowledge, no comparable study has been reported, relating plasma retinol with SRH. Similarly, we did not find studies assessing *α*-tocopherol and SRH, for which we could not find a significant association in the current analysis. A recent study, however, assessed the effects of vitamins on mortality, for which vitamin A, together with other vitamins (B_2_, B_6_, C, E and folic acid) and Fe were associated with a lower risk of mortality (hazard ratio 0·69, 95 % CI 0·48, 0·99)^(40)^. Moreover, a study based on the Danish WHO MONICA surveys associated poor SRH with diets in adults lacking adequate intakes of fruit and vegetables (sources of vitamins A and E)^(20)^.

Overall, several features differed significantly in participants from the categories of self-perceived health status with the following observed in those in the fair/poor category: apart from having lower vitamin A and vitamin D plasma levels, they also tended to be older, have a higher BMI and waist cirumference, a higher percentage were smokers, were taking medications and had co-morbidities. They also had a higher rate of hospitalisations in the year preceding participation in the study. Unexpectedly, the percentage of alcohol abstainers was higher; however, this might be explained by the fact that they no longer consumed alcohol as a result of poorer health, or because we categoriesed the cohort into those who consumed *v*. those who did not consume alcohol, and thus might have overlooked the suggested benefits of moderate alcohol consumption^(41)^. Logistic regression analyses controlled for the above confounders with the advantage of assessing the four different categories of SRH (as compared with binary logistic regression, which collapsed them into two categories only) and some of these variables demonstrated a significantly higher odds of reporting fair/poor health status: BMI, smoking status, season, education and marital status, number of medications taken, number of co-morbidities and hospitalisations in the past year, and country. These findings corroborate previously reported associations to health status^(34,42)^.

With regard to pathophysiological links between low plasma status of the fat-soluble micronutrients A and D and health, inadequate intake is one likely causal factor^(8,12)^. A recent study in the USA using data from the 2005–2016 NHANES demonstrated that 45 %, 95 % and 84 % of the population were below the estimated average requirement for vitamins A, D and E, respectively^(17)^. A comparable analysis displayed slightly lower values in Germany, where for example, a national survey reported the following percentage of adults to display inadequate intakes of fat-soluble vitamins (results are reported separately for men and women, respectively): vitamin A: 15 % and 10 %; vitamin D: 82 % and 91 %; vitamin E: 48 % and 49 %^(43)^. Nutritional status, however, is influenced by other factors other than nutritional intake. For example, vitamin D is primarily obtained from sun exposure and this plays a critical role in vitamin D status, as does the season during which blood is sampled^(18)^. Furthermore, serum 25-hydroxvitamin D levels naturally decline during the ageing process due to a reduction in the cutaneous production of vitamin D and renal production of 1,25-dihydroxyvitamin D^(44)^. Such a reduction is also observed for vitamins A and E. In our study, *α*-tocopherol levels did appear to differ significantly between the four age groups, but the values increased with increasing age whereas they remained stable for vitamin D (data not shown). Drug–nutrient interactions can also not be ruled out as playing a causative role, as discussed by Weber *et al*.^(45)^ Recently, indications of bidirectional interactions between fat-soluble vitamins and the gut microbiome at a molecular and functional level have been reported, with the former affecting gut microbial composition and a dysbiotic microbiome exerting effects on the status, metabolism and function of these vitamins^(46)^. Mechanisms of actions include modulation of the vitamin D and retinoic acid receptors, vitamin transport systems, thus hindering absorption efficiency, and the biotransformation pathways^(46)^.

The current study is limited by its cross-sectional design because no inference could be made regarding the time lag between micronutrient status and SRH. Blood sampling for the plasma concentrations of the fat-soluble vitamins were taken at one time point only, thus might not be reflective of the respective nutrient status preceding study participation. A recent study noted that SRH can predict mortality with a time-dependent effect^(47)^. This concept might also pertain to micronutrient status and subsequent health status rating. Moreover, from our analysis, one cannot ascertain whether the reported lipophilic micronutrient concentrations observed are a cause or a consequence of the respective SRH. For example, poor SRH will likely also worsen low serum 25-hydroxyvitamin D concentrations, as people who feel unwell might be more reluctant to spend time outdoors. As such vitamin D status might simply reflect a person’s way of life, where the two conditions exacerbate each other. A recent study, however, reported the effects of vitamin D on SRH to persist even when adjusting for chronic conditions and including healthy participants only^(15)^. The lack of data on physical activity and household income are also confounders that were not controlled for.

Our results might not be applicable to other study polulations, since we included a group of participants (the GO group) that were offspring of nonagenarians and thus are considered genetically priviledged. In this regard, the GO group had the highest percentage of participants rating health status as excellent. Also, for the present analysis, data from participants taking supplements were included as a predictor variable, but the type of supplements taken was unknown. We nevertheless deemed it valuable to include these participants, since our main outcome was assessing effects of micronutrient status in plasma rather than nutritional intake and because supplement use as been positively associated with SRH in previous studies^(48)^. The large sample size is a strength of our study, as is the SRH (the main outcome) which has been reported to accurately reflect objective health status and thus has led to its recommendation as a global complementary measure of health^(49)^.

Overall, these findings suggest that fat-soluble vitamins A and D might be markers of health status. Interventions to improve plasma concentrations of these nutrients could therefore be beneficial in those with fair/poor SRH. Steptoe *et al.* ^(50)^ reported improvements to SRH after 12 months of an intervention increasing fruit and vegetable consumption, reflected by increases in plasma micronutrient status^(51)^. A meta-analysis demonstrated that people with poor SRH have a twofold higher mortality risk than those with excellent SRH^(42)^; therefore, such targeted interventions for people identified as having fair/poor SRH could provide an opportunity for nutrient status modifications.

### Conclusion

Multiple logistic regression identified both lower plasma vitamin A and D status as being independently associated with fair/poor SRH. Since poorer SRH has been associated with increased mortality, even in the absence of chronic disease, further insights into the long-term implications of these modifiable nutrients on health status are warranted.

## Supplementary material

For supplementary material accompanying this paper visit https://doi.org/10.1017/S0007114521004633. click here to view supplementary material
