# Supplementary material for: Association between fat-soluble vitamins and self-reported health status: a cross-sectional analysis of the MARK-AGE cohort
Source: Br J Nutr. 2021 Nov 19;128(3):433–43. doi: 10.1017/S0007114521004633 (PMC9340855; doi:10.1017/S0007114521004633)
Supplement: Supplementary file 1 [file S0007114521004633sup001.docx]

**Association between fat-soluble vitamins and self-reported health status: a cross-sectional analysis of the MARK-AGE cohort – Supplementary material**

Caroline S. Stokes^1,2,^*, Daniela Weber^1,3^, Stefan Wagenpfeil^4^, Wolfgang Stuetz^5^, María Moreno-Villanueva^6,7^, Martijn E. T. Dollé^8^, Eugène Jansen^8^, Efstathios S. Gonos^9^, Jürgen Bernhardt^10^, Beatrix Grubeck-Loebenstein^11^, Simone Fiegl^12^, Ewa Sikora^13^, Olivier Toussaint^14^§, Florence Debacq-Chainiaux^14^, Miriam Capri^15,16^, Antti Hervonen^17^, P. Eline Slagboom^18^, Nicolle Breusing^19^, Jan Frank^5^, Alexander Bürkle^6^, Claudio Franceschi^20^, Tilman Grune^1,3,21,22,23,24^

^1^ Department of Molecular Toxicology, German Institute of Human Nutrition, 14558 Potsdam-Rehbrücke, Germany

^2^ Food and Health Research Group, Faculty of Life Sciences, Humboldt-Universität zu Berlin, 14195 Berlin, Germany

^3^ NutriAct-Competence Cluster Nutrition Research Berlin-Potsdam, Nuthetal 14458, Germany

^4^ Institute of Medical Biometry, Epidemiology and Medical Informatics, Saarland University, Homburg, Germany

^5^ Department of Food Biofunctionality, Institute of Nutritional Sciences (140), University of Hohenheim, 70599 Stuttgart, Germany

^6^ Molecular Toxicology Group, Department of Biology, University of Konstanz, 78457 Konstanz, Germany

^7^ Human Performance Research Centre, Department of Sport Science, University of Konstanz, 78457 Konstanz, Germany

^8^ Centre for Health Protection, National Institute for Public Health and the Environment, PO Box 1, 3720 BA Bilthoven, The Netherlands

^9^ National Hellenic Research Foundation, Institute of Biology, Medicinal Chemistry and Biotechnology, Athens, Greece

^10^ BioTeSys GmbH, Schelztorstr. 54-56, 73728 Esslingen, Germany

^11^ Research Institute for Biomedical Aging Research, University of Innsbruck, Rennweg, 10, 6020 Innsbruck, Austria

^12^ UMIT TIROL – Private University for Health Sciences, Medical Informatics and Technology, 6060 Hall in Tyrol, Austria

^13^ Laboratory of the Molecular Bases of Ageing, Nencki Institute of Experimental Biology, Polish Academy of Sciences, 3 Pasteur street, 02-093 Warsaw, Poland

^14^ URBC-NARILIS, University of Namur, Rue de Bruxelles, 61, Namur, Belgium

^15^ Department of Experimental, Diagnostic and Specialty Medicine, Alma Mater Studiorum, University of Bologna, Bologna, Italy.

^16^ Interdepartmental Center - Alma Mater Research Institute on Global Challenges and Climate Change - University of Bologna, Bologna, Italy

^17^ Medical School, University of Tampere, 33014 Tampere, Finland

^18^ Section of Molecular Epidemiology, Leiden University Medical Centre, Leiden, The Netherlands

^19^ Department of Applied Nutritional Science/Dietetics, Institute of Nutritional Medicine, University of Hohenheim, Stuttgart 70599, Germany

^20^ Department of Experimental Pathology, University of Bologna, Bologna, Italy

^21^ German Center for Diabetes Research (DZD), 85764 München-Neuherberg, Germany

^22^ German Center for Cardiovascular Research (DZHK), Partner Site Berlin, 13347 Berlin, Germany

^23^ University of Potsdam, Institute of Nutritional Science, Nuthetal, Germany

^24^ University of Vienna, Department of Physiological Chemistry, Faculty of Chemistry, 1090 Vienna, Austria

^§^ deceased

^*^Correspondence: Prof. Dr. Caroline Stokes,

Department of Molecular Toxicology, Nutrients and Health Research Group

German Institute of Human Nutrition, 14558 Potsdam-Rehbrücke, Germany

Phone: +49 151 535 83778; Email: [caroline.stokes@dife.de](mailto:caroline.stokes@dife.de)

**Analytical Methods**

Given the larger variation in age range for the RASIG group (35–75 years) compared to the GO and SGO groups (mainly 55–75 years), subgroup analyses stratified by age with participants ≥55 years only were carried out to compare health status (depicted in the flow chart below).

**Flow Chart:** Flow Chart depicting the main analyses for the overall cohort in addition to the subgroup analysis based on a cut-off of 55 years of age

**
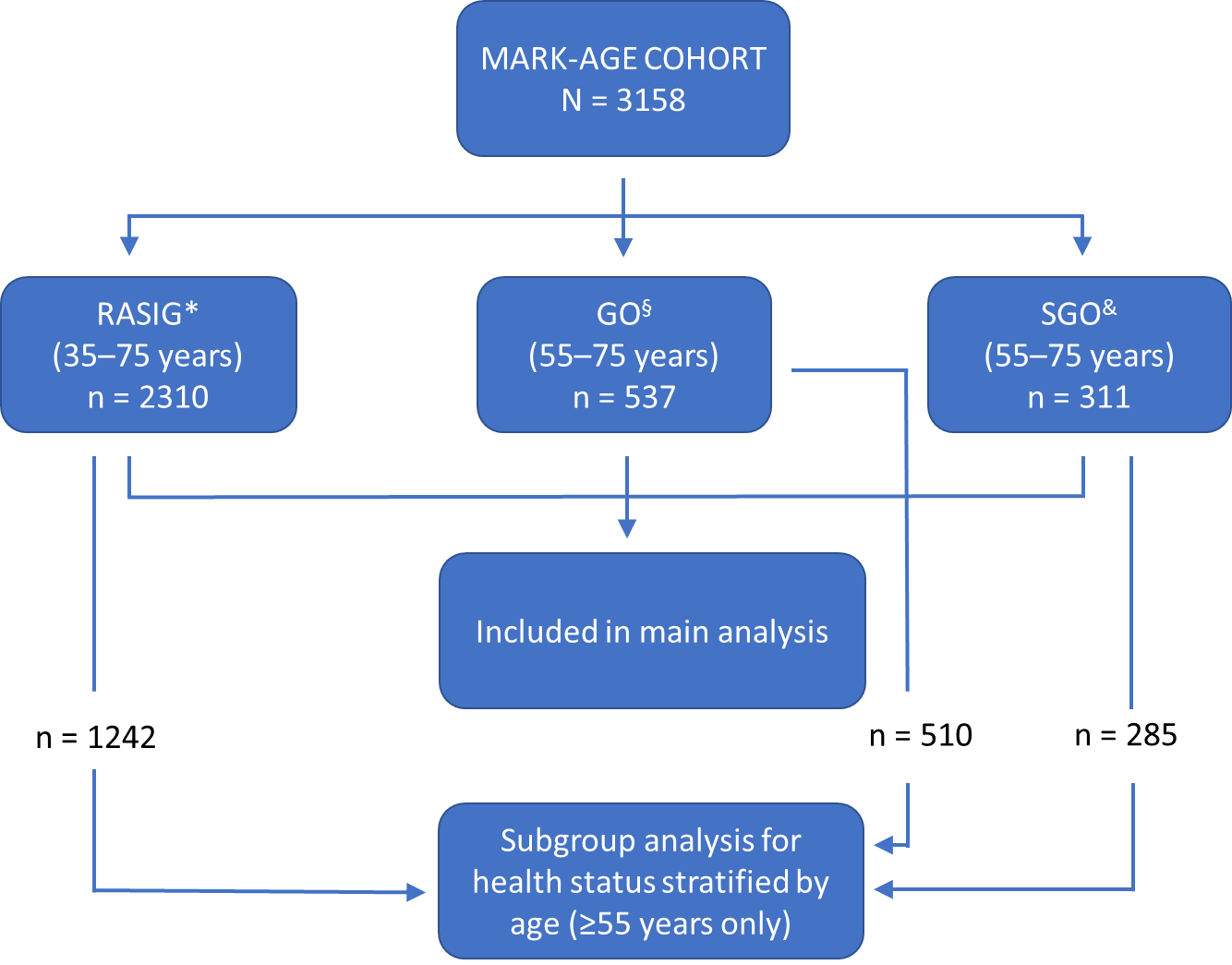
**

*RASIG group (age-stratified individuals from the general population)

^§^GO group (Genetics of Healthy Aging GEHA offspring)

^&^SGO group (spouses from the recruited GO participants)

**Supplementary Table 1: Self-rated health status based on study group**

|  | **RASIG** | **GO** | **SGO** |
| --- | --- | --- | --- |
| ***Health Status*** | (n=2310) | (n=537) | (n=311) |
| Excellent | 267 (11.6%) | 76 (14.2%) | 26 (8.4%) |
| Very good | 880 (38.1%) | 152 (28.3%) | 80 (25.7%) |
| Good | 897 (38.8%) | 236 (43.9%) | 159 (51.1%) |
| Fair/poor | 266 (11.5%) | 73 (13.6%) | 46 (14.8) |
| **≥ 55 years old** | **RASIG** | **GO** | **SGO** |
| ***Health Status*** | (n=1242) | (n=510) | (n=285) |
| Excellent | 107 (8.6) | 75 (14.7) | 23 (8.1) |
| Very good | 418 (33.7) | 143 (28.0) | 70 (24.6) |
| Good | 519 (41.8) | 221 (43.3) | 146 (51.2) |
| Fair/poor | 198 (15.9) | 71 (13.9) | 46 (16.1) |

The groups of study participants: RASIG (73.1%); GO (17.1%); SGO (9.8%) differed significantly based on the categories of self-rated health status (χ2(4)=41.49, P<0.001). The association was considered small (Cramer's V=0.081). The GO group had the highest percentage of participants rating health status as excellent (14.2%) and the SGO had the highest percentage for self-reported fair/poor health status (14.8%). Given, that the RASIG group included participants from the age of 35 years, a subgroup comparison between the three groups only including participants ≥55 years confirmed the above findings (χ2(4)=28.53, P<0.001; Cramer’s V=0.84).

**Supplementary Table 2: Self-rated health status according to country**

|  | **Austria** | **Belgium** | **Finland** | **Germany** | **Greece** | **Italy** | **Netherlands** | **Poland** |
| --- | --- | --- | --- | --- | --- | --- | --- | --- |
| ***Health Status*** | (n=399) | (n=379) | (n=294) | (n=357) | (n=425) | (n=563) | (n=215) | (n=526) |
| Excellent | 81 (20.3%) | 60 (15.8%) | 42 (14.3%) | 45 (12.6%) | 56 (13.2%) | 44 (7.8%) | 38 (17.7%) | 3 (0.6%) |
| Very good | 205 (51.4%) | 147 (38.8%) | 109 (37.1%) | 153 (42.9%) | 160 (37.6%) | 178 (31.6%) | 67 (31.2%) | 93 (17.7%) |
| Good | 107 (26.8%) | 133 (35.1%) | 97 (33.0%) | 139 (38.9%) | 144 (33.9%) | 285 (50.6%) | 98 (45.6%) | 289 (54.9%) |
| Fair/poor | 6 (1.5%) | 39 (10.3) | 46 (15.6%) | 20 (5.6%) | 65 (15.3) | 56 (9.9) | 12 (5.6%) | 141 (26.8%) |
| **≥ 55 years old** | **Austria** | **Belgium** | **Finland** | **Germany** | **Greece** | **Italy** | **Netherlands** | **Poland** |
| ***Health Status*** | (n=200) | (n=273) | (n=258) | (n=198) | (n=218) | (n=349) | (n=212) | (n=329) |
| Excellent | 30 (15.0) | 43 (15.8) | 33 (12.6) | 23 (11.6) | 19 (8.7) | 19 (5.4) | 37 (17.5) | 1 (0.3) |
| Very good | 101 (50.5) | 92 (33.7) | 93 (36.0) | 85 (42.9) | 65 (29.8) | 91 (26.1) | 67 (31.6) | 37 (11.2) |
| Good | 65 (32.5) | 104 (38.1) | 88 (34.1) | 79 (39.9) | 83 (38.1) | 191 (54.7) | 96 (45.3) | 180 (54.7) |
| Fair/poor | 4 (2.0) | 34 (12.5) | 44 (17.1) | 11 (5.6) | 51 (23.4) | 48 (13.8) | 12 (5.7) | 111 (33.7) |

The highest percentage of participants reporting fair/poor health status was from Poland (26.8% in all ages; 33.7% in those ≥55 years), with the lowest reported from Austria with 1.5% (2% in ≥55 years). Health status category was statistically significantly associated with country (χ2(21)=421.58, P<0.001) with a moderate to small association (Cramer's V=0.21). Similar results were obtained when stratifying the cohort according to age for those 55 years and older (χ2(21)=313.41, P<0.001; Cramer's V=0.23).

**Supplementary Table 3: Univariate binary logistic regression analysis**

For binary logistic regression the dependent variable, health status categories were dichotomised as follows: excellent/very good/good versus fair/poor. The univariate analysis assessed vitamin D, retinol and α-tocopherol as well as the following confounders: sex, age group, education, marital status, BMI, smoking and alcohol consumption status, supplement use, number of current comorbidities, number of medications, hospital visits during the preceding 12 months, season of blood sampling, vitamin D status, quartiles for retinol and α-tocopherol, country of residence and study group.

The following variables contained missing values (ordered as presented in table): BMI (n=1), Hospitalisation status (n=6), 25-hydroxyvitamin D (n=140), α-tocopherol (n=136), retinol (n=136).

|  | **OR (95% CI)** | **P** |
| --- | --- | --- |
|  |  |  |
| Sex: Male | 0.916 (0.740, 1.135) | 0.42 |
| Age group: 35-44 | Reference | < 0.001 |
| Age group 45-54 | 2.222 (1.294, 3.815) | 0.004 |
| Age group 55-64 | 4.185 (2.563, 6.834) | < 0.001 |
| Age group 65-75 | 5.005 (3.073, 8.150) | < 0.001 |
| Education: University degree | Reference | < 0.001 |
| Finished school | 1.753 (1.359, 2.260) | < 0.001 |
| Elementary unfinished | 5.232 (2.386, 11.473) | < 0.001 |
| Married | Reference | 0.006 |
| Never | 0.878 (0.602, 1.281) | 0.49 |
| Divorced | 1.386 (1.025, 1.874) | 0.034 |
| Widow | 1.768 (1.191, 2.623) | 0.005 |
| BMI (kg/m2) | 1.106 (1.083, 1.130) | < 0.001 |
| Supplements: yes | 0.856 (0.689, 1.063) | 0.16 |
| Alcohol consumption: yes | 0.455 (0.352, 0.589) | < 0.001 |
| Smoker: no | Reference | < 0.001 |
| Smoker: current | 1.894 (1.437, 2.497) | < 0.001 |
| Smoker: previous | 1.253 (0.980, 1.601) | 0.07 |
| Meds (0) | Reference | < 0.001 |
| Meds (1-2) | 1.923 (1.400, 2.639) | < 0.001 |
| Meds (3-4) | 5.713 (4.137, 7.889) | < 0.001 |
| Meds (5+) | 11.166 (7.999, 15.589) | < 0.001 |
| Comorbidities (0-1) | Reference | < 0.001 |
| Comorbidities (2-3) | 1.994 (1.320, 3.012) | < 0.001 |
| Comorbidities (4+) | 10.062 (6.992, 14.481) | < 0.001 |
| Hospitalised past 12 mo: yes | 2.193 (1.658, 2.901) | < 0.001 |
| Summer | Reference | < 0.001 |
| Spring | 1.577 (1.120, 2.220) | 0.009 |
| Winter | 1.993 (1.408, 2.820) | < 0.001 |
| Autumn | 2.320 (1.633, 3.294) | < 0.001 |
| Optimal vitamin D | Reference | < 0.001 |
| Insufficient vitamin D | 1.604 (0.953, 2.700) | 0.075 |
| Vitamin D deficiency | 2.636 (1.596, 4.354) | < 0.001 |
| Severe vitamin D deficiency | 4.153 (2.419, 7.130) | < 0.001 |
| α-Tocopherol quartiles (µmol) >33.37 | Reference | 0.38 |
| 28.22-33.37 | 1.012 (0.733, 1.399) | 0.941 |
| 23.85-28.21 | 1.183 (0.864, 1.620) | 0.294 |
| <23.85 | 1.256 (0.921, 1.713) | 0.150 |
| Retinol quartile (µmol) (>2.02) | Reference | 0.017 |
| 1.73-2.02 | 0.917 (0.661, 1.272) | 0.604 |
| 1.45-1.72 | 1.019 (0.743, 1.398) | 0.91 |
| <1.45 | 1.438 (1.063, 1.945) | 0.02 |
| Germany | Reference | < 0.001 |
| Finland | 3.125 (1.803, 5.417) | < 0.001 |
| Belgium | 1.933 (1.104, 3.383) | 0.021 |
| Netherlands | 0.996 (0.477, 2.981) | 0.99 |
| Poland | 6.171 (3.778, 10.079) | < 0.001 |
| Greece | 3.042 (1.804, 5.131) | < 0.001 |
| Austria | 0.257 (0.102, 0.648) | 0.004 |
| Italy | 1.861 (1.097, 3.158) | 0.02 |
| Study group: RASIG | Reference | 0.14 |
| GO | 1.209 (0.916, 1.596) | 0.18 |
| SGO | 1.334 (0.951, 1.870) | 0.09 |

**Supplementary Table 4: Univariate ordinal logistic regression analysis**

Ordinal logistic regression was performed to determine the effect of the three fat-soluble vitamins on the four groups of SRH (excellent, very good, good, fair/good) as the dependent variable taking into account possible confounders. The following predictor variables were assessed in a univariate analysis: sex, age group, education, marital status, BMI, smoking and alcohol consumption status, supplement use, number of current comorbidities, number of medications, hospital visits during the preceding 12 months, season of blood sampling, vitamin D status, quartiles for retinol and α-tocopherol, country of residence and study group.

The following variables contained missing values (ordered as presented in table): BMI (n=1), Hospitalisation status (n=6), 25-hydroxyvitamin D (n=140), α-tocopherol (n=136), retinol (n=136).

|  |  | **OR (95% CI)** | **P** |
| --- | --- | --- | --- |
|  |  |  |  |
| Sex: Male |  | 0.834 (0.734, 0.949) | 0.006 |
| Age group: 35-44 |  | Reference | < 0.001 |
| Age group 45-54 |  | 1.153 (0.981, 1.354) | 0.08 |
| Age group 55-64 |  | 1.686 (1.401, 2.029) | < 0.001 |
| Age group 65-75 |  | 2.749 (2.247, 3.364) | < 0.001 |
| Education: University degree |  | Reference | < 0.001 |
| Finished school |  | 2.222 (1.145, 4.313) | 0.02 |
| Elementary unfinished |  | 3.386 (1.735, 6.608) | < 0.001 |
| Married |  | Reference | 0.004 |
| Never |  | 1.578 (1.194, 2.087) | 0.001 |
| Divorced |  | 1.636 (1.172, 2.284) | 0.004 |
| Widow |  | 1.306 (0.945, 1.805) | 0.11 |
| BMI (kg/m2) |  | 0.911 (0.897, 0.924) | < 0.001 |
| Supplements: yes |  | 0.980 (0.859, 1.119) | 0.76 |
| Alcohol consumption: yes |  | 0.496 (0.412, 0.597) | < 0.001 |
| Smoker: no |  | Reference | < 0.001 |
| Smoker: previous |  | 1.325 (1.093, 1.608) | 0.004 |
| Smoker: current |  | 1.834 (1.527, 2.202) | < 0.001 |
| Meds (0) |  | Reference | < 0.001 |
| Meds (1-2) |  | 1.898 (1.435, 2.512) | < 0.001 |
| Meds (3-4) |  | 4.962 (3.854, 6.389) | < 0.001 |
| Meds (5+) |  | 8.492 (6.618, 10.897) | < 0.001 |
| Comorbidities (0-1) |  | Reference | < 0.001 |
| Comorbidities (2-3) |  | 3.487 (2.954, 4.116) | < 0.001 |
| Comorbidities (4+) |  | 6.330 (5.319, 7.533) | < 0.001 |
| Hospitalised past 12 mo: yes |  | 1.806 (1.470, 2.219) | < 0.001 |
| Summer |  | Reference | < 0.001 |
| Spring |  | 1.194 (0.987, 1.446) | 0.07 |
| Winter |  | 1.406 (1.173, 1.684) | < 0.001 |
| Autumn |  | 1.553 (1.275, 1.892) | < 0.001 |
| Optimal vitamin D |  | Reference | < 0.001 |
| Insufficient vitamin D |  | 1.404 (1.131, 1.744) | 0.002 |
| Vitamin D deficiency |  | 1.864 (1.494, 2.327) | < 0.001 |
| Severe vitamin D deficiency |  | 2.903 (2.192, 3.844) | < 0.001 |
| α-Tocopherol quartiles (µmol) >33.37 |  | Reference | 0.48 |
| 28.22-33.37 |  | 0.927 (0.770, 1.116) | 0.42 |
| 23.85-28.21 |  | 1.074 (0.892, 1.292) | 0.45 |
| <23.85 |  | 1.022 (0.849, 1.230) | 0.82 |
| Retinol quartile (µmol) (>2.02) |  | Reference | < 0.001 |
| 1.73-2.02 |  | 1.381 (1.147, 1.664) | 0.001 |
| 1.45-1.72 |  | 1.498 (1.240, 1.809) | < 0.001 |
| <1.45 |  | 1.426 (1.183, 1.719) | < 0.001 |
| Germany |  | Reference | < 0.001 |
| Finland |  | 1.286 (0.966, 1.711) | 0.09 |
| Belgium |  | 0.721 (0.554, 0.937) | 0.014 |
| Netherlands |  | 2.387 (1.802, 3.162) | < 0.001 |
| Poland |  | 0.971 (0.737, 1.280) | 0.83 |
| Greece |  | 0.257 (0.196, 0.337) | < 0.001 |
| Austria |  | 1.183 (0.854, 1.638) | 0.31 |
| Italy |  | 1.242 (0.937, 1.647) | 0.13 |
| Study group: RASIG |  | Reference | < 0.001 |
| GO |  | 1.381 (1.066, 1.789) | 0.014 |
| SGO |  | 1.663 (1.335, 2.072) | < 0.001 |
